# Prevalence, Distribution, and Phylogeny of Type Two Toxin-Antitoxin Genes Possessed by *Cronobacter* Species where *C. sakazakii* Homologs Follow Sequence Type Lineages

**DOI:** 10.3390/microorganisms7110554

**Published:** 2019-11-12

**Authors:** Samantha Finkelstein, Flavia Negrete, Hyein Jang, Jayanthi Gangiredla, Mark Mammel, Isha R. Patel, Hannah R. Chase, JungHa Woo, YouYoung Lee, Caroline Z. Wang, Leah Weinstein, Ben D. Tall, Gopal R. Gopinath

**Affiliations:** Center of Food Safety and Applied Nutrition, U. S. Food and Drug Administration, 8301 MuirKirk Rd, Laurel, MD 20708, USA; sfinkel6@terpmail.umd.edu (S.F.); Flavia.Negrete@fda.hhs.gov (F.N.); hyein.jang@fda.hhs.gov (H.J.); jayanthi.gangiredla@fda.hhs.gov (J.G.); mark.mammel@fda.hhs.gov (M.M.); isha.patel@fda.hhs.gov (I.R.P.); hannahchase11@yahoo.com (H.R.C.); junghaa12@gmail.com (J.W.); luy902@naver.com (Y.L.); caroline.z.wang@duke.edu (C.Z.W.); lmweinstein1@gmail.com (L.W.); Gopal.GOPINATHRAO@fda.hhs.gov (G.R.G.)

**Keywords:** toxin–antitoxins, *Cronobacter*, microarray, phylogeny

## Abstract

*Cronobacter* species are a group of foodborne pathogenic bacteria that cause both intestinal and systemic human disease in individuals of all age groups. Little is known about the mechanisms that *Cronobacter* employ to survive and persist in foods and other environments. Toxin–antitoxin (TA) genes are thought to play a role in bacterial stress physiology, as well as in the stabilization of horizontally-acquired re-combinatorial elements such as plasmids, phage, and transposons. TA systems have been implicated in the formation of a persistence phenotype in some bacterial species including *Escherichia coli* and *Salmonella*. This project’s goal was to understand the phylogenetic relatedness among TA genes present in *Cronobacter*. Preliminary studies showed that two typical toxin genes, *fic* and *hipA* followed species evolutionary lines. A local database of 22 TA homologs was created for *Cronobacter sakazakii* and a Python version 3 shell script was generated to extract TA FASTA sequences present in 234 *C. sakazakii* genomes previously sequenced as part of Center for Food Safety and Applied Nutrition’s (CFSAN) GenomeTrakr project. BLAST analysis showed that not every *C. sakazakii* strain possessed all twenty-two TA loci. Interestingly, some strains contained either a toxin or an antitoxin component, but not both. Five common toxin genes: ESA_00258 (*parDE* toxin-antitoxin family), ESA_00804 (*relBE* family), ESA_01887 (*relBE* family), ESA_03838 (*relBE* family), and ESA_04273 (*YhfG-Fic* family) were selected for PCR analysis and the primers were designed to detect these genes. PCR analysis showed that 55 of 63 strains possessed three of these genes Sequence analysis identified homologs of the target genes and some of the strains were PCR-negative for one or more of the genes, pointing to potential nucleotide polymorphisms in those loci or that these toxin genes were absent. Phylogenetic studies using a *Cronobacter* pan genomic microarray showed that for the most part TAs follow species evolutionary lines except for a few toxin genes possessed by some *C. malonaticus* and *C. universalis* strains; this demonstrates that some TA orthologues share a common phylogeny. Within the *C. sakazakii* strains, the prevalence and distribution of these TA homologs by *C. sakazakii* strain BAA-894 (a powdered infant formula isolate) followed sequence-type evolutionary lineages. Understanding the phylogeny of TAs among the *Cronobacter* species is essential to design future studies to realize the physiological mechanisms and roles for TAs in stress adaptation and persistence of *Cronobacter* within food matrices and food processing environments.

## 1. Introduction

Toxin–antitoxin (TA) systems are small genetic elements found on plasmids, phage genomes, and chromosomes of many bacterial and archaeal species [1,2,3,4]. They are usually observed as bi-cistronic gene pairs with the antitoxin gene preceding the toxin gene. In general, the toxin component is a stable protein that inhibits cellular processes, and the antitoxin is an unstable protein that directly binds to its associated toxin or mRNA transcript, which allows for physiological processes to continue by homeostatic means. When bacteria encounter a stressful environment, the antitoxin gets degraded by host proteases, such as Lon and ClpXP; which releases the toxin to bind to mRNA transcripts, halting essential transcription of key genes involved in the physiological processes like transcription/translation of critical cellular processes, cell division, DNA replication, ATP synthesis, mRNA stability, or cell wall synthesis [5].

Bacterial TA systems were initially discovered through their involvement in maintaining plasmids [4], and subsequently these plasmids’ role in antibiotic resistance. It has been posited that plasmidborne toxin/antitoxin loci serve only to maintain plasmid DNA at the expense of the host organism, while studies reported by other authors suggest that plasmidborne TAs maintain plasmids for host competitive advantages [6]. Furthermore, like plasmidborne TAs, chromosomal TAs might play a role in stabilizing genomic and pathogenicity islands and prophages, which are similarly obtained through horizontal gene transfer mechanisms [6,7]. In fact, it is thought that the prophage P1 constitutes a low copy number plasmid through its lysogeny into the host cell’s chromosome [7]. It has been conjectured that during antibiotic therapy, a small population of cells are able to survive antibiotic treatment by entering a transient state of dormancy or persistence. However, a recent report by Goormaghtigh et al. [8] showed that the direct link between induction of type II TA systems and persistence brought on by antibiotics might not be as straightforward as was once thought in these cells.

To date, there are six known classes, or types of TA systems [5]. Type I and Type III TA systems have antitoxins that are small, non-coding RNAs while Type II, IV, V, and VI TA systems have antitoxins that are proteins. Type I toxin–antitoxin systems depend on sequence homology of the complementary antitoxin RNA with the toxin mRNA. Translation of the mRNA is then prevented either through degradation by means of RNase III activity or by obstructing the Shine-Dalgarno sequence (or ribosome binding site) of the toxin mRNA. Type III antitoxins inhibit their cognate toxins through protein-RNA interactions. Type II antitoxins neutralize the harmful effects of their toxins by directly binding to them. Type IV antitoxins prevent their toxin from binding to cytoskeleton proteins. The Type V antitoxin is an endoribonuclease, which cleaves the mRNA of the toxin to halt its expression. Type VI TAs only occur in microorganisms rich in AT complexes, and its toxin blocks the beta sliding clamp, preventing DNA synthesis, which can be selectively targeted by proteases [2,9].

Even though the role of TAs in persistence of cells during antibiotic therapy is now controversial, these genes have not been well-characterized in the *Cronobacter* species [10,11]. *Cronobacter* species are Gram-negative bacteria that are opportunistic foodborne pathogens that cause intestinal and systemic human disease [12,13]. The genus *Cronobacter* taxonomically consists of seven species including *Cronobacter sakazakii*, *Cronobacter malonaticus*, *Cronobacter turicensis*, *Cronobacter muytjensii*, *Cronobacter universalis, Cronobacter dublinensis* (with three subspecies—*C. dublinensis* subsp. *dublinensis*, *C. dublinensis* subsp. *lausannensis,* and *C. dublinensis* subsp. *lactaridi*) and *Cronobacter condimenti* [12,13,14]. *Cronobacter* species were once thought to only infect neonates [15,16,17]. *Cronobacter* are now known to infect individuals of all ages [18,19,20,21]. The species considered most pathogenic include *C. sakazakii, C. malonaticus*, and *C. turicensis*, causing necrotizing enterocolitis, bacteremia, and meningitis in neonates and infants and pneumonia, septicemia, and urinary tract infections in adults [16,18,19,21,22,23,24]. However, all species of *Cronobacter*, except for *C. condimenti* have been associated with infections. Infantile infections are thought to occur through consumption of intrinsic or extrinsic contamination of reconstituted, temperature-abused powdered infant formula (PIF) [23,25]. Jason reported surveillance information on over 80 infant *Cronobacter* cases (which occurred between 1958 and 2010 and is defined here as a confirmed culture-positive case of septicemia or meningitis) where infants exclusively ingested breast milk, without consumption of a commercially manufactured PIF product, prior to illness onset [17]. Friedemann had also reported similar earlier observations [26]. More recently, Bowen et al. [27] and McMullan et al. [28] described cases involving infantile *C. sakazakii* septicemia/meningitis infections where infants had only consumed expressed maternal milk (EMM) during the first weeks after birth. Contaminated personal breast pumps were found to be the source of contamination. Further epidemiological investigations using whole genome sequencing (WGS) analysis determined that the clinical isolates obtained during the investigation were indistinguishable from those cultured from a contaminated breast pump and a home kitchen sink drain in the first case and a breast pump in the latter case. Of equal importance is that *Cronobacter* species are now known to be largely more ecologically prevalent than was once believed, and many researchers have reported their isolation from many types of foods besides PIF and follow-up-formulas, including other low water activity foods, such as dried milk protein products, cereals, cheeses (and cheese powders), licorice, candies, spices, teas, nuts, herbs, and pastas [13,29,30,31,32]. It has also been found to be associated with a variety of ready-to-eat vegetables, body surfaces, and intestinal contents of filth and stable flies, environments of PIF or dairy powder production facilities and households, household sink drains, and water, which might help explain how infections occur in adults [33,34,35,36,37,38,39,40,41,42]. Recently, Yong et al. reported an epidemiological investigation of an acute gastroenteritis outbreak in a high school caused by a mixture of sequence types (ST) ST73 and ST4 *C. sakazakii* strains and ST567 *C. malonaticus* strains. The clinical, implicated food, and environmental samples included rectal swabs, a bean curd braised pork dish, and food delivery boxes [43]. This is the first report of a foodborne gastroenteritis outbreak caused by *Cronobacter* strains involving young teens and adults.

Identification of TA genes is critical for a reliable estimation of gene function. Understanding the genomic landscape including the phylogenetic relatedness of TA genes among *Cronobacter* spp. is essential for designing future studies to identify mechanisms used for survival and persistence in foods. Furthermore, more information about the mechanisms of *Cronobacter* adaptation in a wide range of environments, could eventually lead to development of methods to control the contamination of foods by these organisms. This study establishes the first report describing the phylogeny, prevalence, and distribution of various type two toxin–antitoxin genes possessed by *Cronobacter* species in general, and specifically focuses on the prevalence, distribution, and phylogeny of type II TAs possessed by *C. sakazakii* strains. The predictive results described in this report lay the foundation for future functional genetic studies designed to understand the role of these genes in the control of the contamination of foods. 

## 2. Materials and Methods

### 2.1. Bacterial Strains 

The strains described in this report are shown in Table 1 and in Table S1. These strains were obtained through various surveillance studies reported by Restaino et al. [44], Jaradat et al. [45], Chon et al. [46], Yan et al. [47], Gopinath et al. [32], Chase et al. [48], and Jang et al. [40,41]. All strains, except for those whose genomes were obtained from the National Center for Biotechnology Information (NCBI) GenBank, were identified as *Cronobacter* using species-specific (*rpoB* and *cgcA*) PCR assays [49,50,51]. Multi-locus sequence typing (MLST) analysis of the strains was carried out following the protocol described by Joseph et al. [52]. Genome assemblies (FASTA) were submitted to the *Cronobacter* MLST website (http://www.pubmlst.org/cronobacter) where the MLST database assigned allelic, sequence type (ST), and clonal complex (CC) profiles for each strain based on a seven-loci MLST scheme [52,53]. All genomes were also submitted to Center for Food Safety and Applied Nutrition’s (CFSAN) Galaxy GenomeTrakr website’s MLST tool (https://galaxytrakr.org/?tool_id=toolshed.g2.bx.psu.edu%2Frepos%2Fiuc%2Fmlst%2Fmlst%2F2.15.1&version=2.15.1&__identifer=b7m1wdw5jjt) for analysis. Taken together, both approaches confirmed the identity of the strains.

### 2.2. DNA Extraction for PCR Assay, Microarray, and Whole Genome Sequencing (WGS)

Frozen stocks of each strain were plated onto *Enterobacter sakazakii* Chromogenic Plating Medium (ESPM; R&F Products, Downers Grove, IL, USA) and were cultured overnight at 37 °C. A single colony of each *Cronobacter* strain displaying the typically blue-black to blue-gray colony on ESPM was inoculated into 5 mL of Trypticase soy broth (BBL, Cockeysville, MD, USA), supplemented with 1% NaCl (TSBS) and then incubated at 37 °C for 18 h with shaking at 150 rpm. Bacterial DNA extraction and purification were performed using a Qiagen QIACube instrument and its automated technology (QIAGEN Sciences, Germantown, MD, USA), as described previously and according to the manufacturer’s instructions [32,41,48,54]. The 109 TA orthologs identified on the pan genomic microarray are described in Table S2.

### 2.3. Microarray Analysis (MA)

The microarray used in this study was an Affymetrix MyGeneChip Custom Array (Affymetrix design number: FDACRONOa520845F), which utilizes the whole genome sequences of 15 *Cronobacter* strains, as well as 18 plasmids. These 15 strains encompassed all currently proposed species of *Cronobacter*. A ≥97% identity threshold level between gene homologs to positively predict allelic coverage was used to obtain genes that were represented on the array as described by Tall et al. [54]. Each gene (19,287 *Cronobacter* gene targets) was represented on the array by 22 unique 25-mer oligonucleotide probes, as described by Tall et al. [54].

### 2.4. Whole Genome Sequencing (WGS), Assemblies, and Annotation for Comparative Genomic Analysis

Two milliliters of an overnight TSBS (total 3 mL volume) culture of each *C. sakazakii* strain was taken, and genomic DNA was extracted using the automated Qiagen QIACube instrument (QIAGEN, Inc., Germantown, MD, USA) as described above. Typical yields of the purified genomic DNA were 5–50 µg from a final elution volume of 200 µL and these were used for the WGS and Microarray analyses. Each strain’s DNA was quantified using a Qubit dsDNA BR assay kit (Invitrogen, Thermo Fisher Scientific, Wilmington, DE, USA) and Qubit 2.0 fluorometer (Life Technologies, Grand Island, NY, USA). The DNA samples were prepared with nuclease-free deionized water (molecular biology grade, Thermo Fisher Scientific, Waltham, MA, USA) with a final concentration of 0.2 ng/μL. Genomic libraries were prepared using the Nextera library prep kit (Illumina, San Diego, CA, USA) and whole genome sequencing was conducted using an Illumina MiSeq platform utilizing either 500 or 600 cycles of paired-end reads.

Raw sequence reads (FASTQ datasets) from the Illumina sequencing were trimmed for removal of adaptor sequences and for quality control purposes, and then de novo assembled using the CLC Genomics Workbench version 9.0 (CLC bio, Aarhus, Denmark). For annotation, the FASTA files of the assemblies were uploaded onto the Rapid Annotation Subsystems Technology (RAST) server (online annotation; http://rast.theseed.org). For routine prokaryotic genome annotation, the files were uploaded using the PGAP pipeline at NCBI. 

Nucleotide sequences of the strains were deposited into NCBI’s GenBank and were released to the public through submission to NCBI under the FDA-CFSAN bioproject *Cronobacter* GenomeTrkr Project (PRJNA258403), which is part of the FDA’s Center for Food Safety and Applied Nutrition (CFSAN) foodborne pathogen research umbrella project at NCBI (PRJNA186875) project. The accession numbers of the genomes used in the study are shown in Table S1.

### 2.5. PCR

Five type II toxin genes and represented TA families—*ESA_00258* (parDE toxin-antitoxin family), *ESA_00804* (relBE family), *ESA_01887* (relBE family), *ESA_03838* (relBE family), *ESA_04273* (YhfG-Fic family) were found in the *C. sakazakii* strain BAA-894 using the PostgreSQL-based TAfinder tool: TADB2 (http://bioinfo-mml.sjtu.edu.cn/TADB2/) [55], and were run in NCBI’s Primer-BLAST so that the PCR primers could be designed to detect these five toxins in other *Cronobacter* strains. Primer sequences and PCR reaction parameters are given in Table 2.

### 2.6. Bioinformatics Analysis: A Database and Local BLAST Analysis

The Type II database located at http://bioinfo-mml.sjtu.edu.cn/TADB2/ was used for screening WGS assemblies. A local database of these assemblies was created and formatted for BLAST analysis. Nucleotide sequences of Type II TAs were obtained from TADB and were confirmed to be present in *C. sakazakii* strain BAA-894 using this local database. Reference genomes were obtained from NCBI and in-house python and Perl scripts were used to parse the BLAST output and identify homologous sequences of TA genes in 234 genomes of *C. sakazakii* strains.

## 3. Results and Discussion

### 3.1. Pan-Genomic Microarray Analysis Demonstrates That Cronobacter TA Allelic Sequence Divergence Aligned along Species Taxa Lines and Within C. sakazakii Aligned with ST Lineages

**Figure 1:**
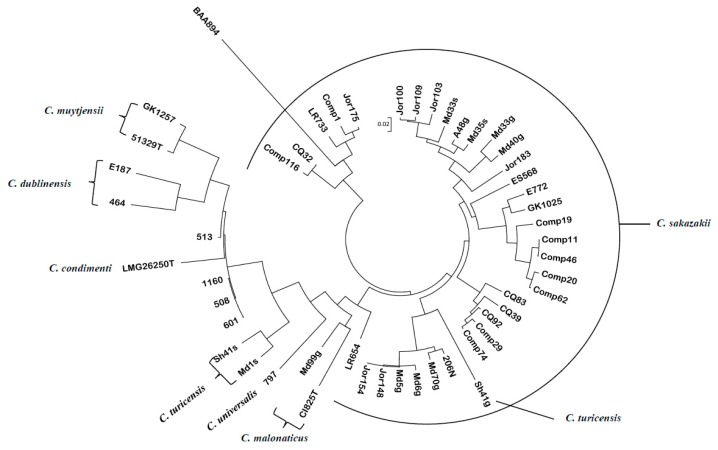
Evolutionary relationships of 50 *Cronobacter* and phylogenetically related strains, which were generated from a gene-difference matrix using just the 109 toxin–antitoxin (TA) probeset homologs shown in Supplementary Table S2. The evolutionary history was inferred using the neighbor-joining method [55]. The microarray experimental protocol, as described by Tall et al. [53], was used for the interrogation of the strains and for the analysis. The optimal tree with the sum of branch length = 2.25204503 is shown. The tree was drawn to scale, with branch lengths in the same units as those of the evolutionary distances used to infer the phylogenetic tree. The evolutionary distances were computed using the p-distance method [56] and are in the units (0.02) of the number of base differences per site. Evolutionary analyses were conducted in MEGA5 [54]. The *Cronobacter* microarray demonstrated that for the most part, the TA allelic sequence divergence aligned along species taxa lines and within *C. sakazakii*, it aligned with ST lineages.

The ~19,000 probe sets comprising the FDA *Cronobacter* custom designed Affymetrix microarray represents the total *Cronobacter* pan genomic content, and for this study the probesets were narrowed down to 109, representing TA orthologues from the seven species [53]. *C. universalis* was only represented by two homologs [FIG00553297: hypothetical protein (ESA_00913) and a putative transcriptional regulator (ESA_01147)]. The twenty-two TA homologs possessed by the *C. sakazakii* strain BAA-894 were confirmed to be present on the *Cronobacter* pan genomic microarray by using BLAST analysis, which compared the TADB sequences with sequences contained in the microarray’s chip design file (Supplemental Table S2). Some of these homologs were represented by TAs from other *C. sakazakii* strains or by TA orthologs of the other species, e.g., TA genes ESA_00803 and ESA_01887 from *C. turicensis;* ESA_00912 from the *C. sakazakii* strain E764; ESA_01146 from the *C. sakazakii* strain Es35; ESA_04371 from *C. sakazakii* strain ES15; and ESA_04372 from *C. condimenti* strain LMG 26250^T^. Two plasmid TA homologs were represented on the microarray as FIG00554131: hypothetical protein (ESA_00912) and was from the *C. sakazakii* strain Es35 and ESA3_p05543 was from the *C. universalis* strain NCTC 9529^T^. There were two phage TA homologs [encoding for YeeV toxin protein and YkfI toxin protein, prophage TAs from the *C. sakazakii* strain 29544^T^] that were represented on the microarray and were from *C. dublinensis* and *C. malonaticus* strain LMG2326^T^. The presence–absence calls generated by the Affymetrix algorithm were brought into MEGA7 [56] to create a phylogenetic tree showing relatedness. Figure 1 represents the results of this analysis and demonstrated that in some but not all species, the TA allelic sequence divergence aligned along the species taxa lines. For example, microarray analysis (MA) showed that the two *C. malonaticus* strains [Md99g, CI825 (LMG2326^T^)] and the *C. universalis* strain NCTC 9529^T^ (797) clustered with some of the *C. sakazakii* strains, signifying that some TAs between these species share a common phylogenetic history. Supplementary Table S2 shows which toxin or antitoxin were present or absent in these strains. Many of these TAs, e.g., the *parDE* family of TA, were also shared by other members of the *Enterobacteriaceae* [5,57].

**Figure 2:**
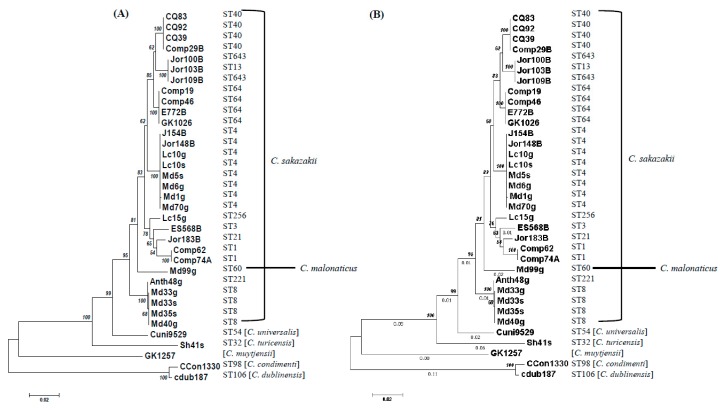
The phylogeny of *fic*-like (**A**) and *hipA* (**B**) toxins were inferred using the neighbor-joining method [55]. The optimal trees with the sum of branch length of 0.38280022 (**A**) and 0.47999136 (**B**) are shown. The trees are drawn to scale, with branch lengths (next to the branches) in the same units as those of the evolutionary distances used to infer the phylogenetic tree. The evolutionary distances were computed using the maximum composite likelihood method [58] and are in the units of the number of base substitutions per site. In both trees, the analysis involved 35 nucleotide sequences. All positions containing gaps and missing data were eliminated. There was a total of 603 positions in (**A**) and 1320 positions in (**B**) in the final dataset. Evolutionary analyses were conducted in MEGA7 [54]. The accession numbers for nucleotide sequences can be found in Table S1.

Preliminary analysis of *fic-like* and *hipA* homologs, two common toxin genes obtained by BLAST analysis from 42 strains representative of the seven species supports the findings obtained by MA. The dendrogram trees shown in Figure 2 demonstrate the relatedness of *fic-like* and *hipA* genes among these *Cronobacter* strains.

The phylogenies based on the sequence divergence of these TA genes, shown in Figure 1 and Figure 2, demonstrate that most strains cluster according to species lineages or in the case of *C. sakazakii*, the strains cluster according to the sequence type. This analysis involved *fic*-*like* and *hipA* type II toxin nucleotide sequences from *C. sakazakii* and single *fic*-*like/hipA* nucleotide sequences, each from *C. malonaticus*, *C. universalis*, *C. turicensis*, *C. muytjensii*, *C. dublinensis*, and *C. condimenti* strains, and each *Cronobacter* species clustered separately. Take note that the *C. sakazakii* strains clustered according to the assigned sequence type designations obtained from (https://pubmlst.org/cronobacter/), as maintained by the University of Oxford [59].

### 3.2. PCR and BLAST Analyses Showed That Not Every C. sakazakii Strain Possessed Similar Numbers or Types of TAs

PCR analysis also exemplified the phylogenetic relatedness of five representative type II TA homologs among *C. sakazakii* strains (the results are summarized in Table 3) and demonstrated that in some strains, TA alleles are shared. PCR results from 63 *C. sakazakii* strains obtained from environmental samples of USA dairy powder manufacturing facilities showed that 55 of the 63 strains (87%) were PCR-positive for these toxin genes (data not shown), suggesting that there were other sequence variations present among the strain’s genomes that were not captured by PCR or that these toxin genes were absent.

Further investigation using the TA sequences identified by local BLAST analysis showed that not every *C. sakazakii* strain possessed all twenty-two TA pairs. The results from this local BLAST analysis of these 22 TA genes against 234 *C. sakazakii* genomes gave an output organized by alleles (the results are summarized in Table 4) and the results of the BLAST analysis for each strain is shown in Supplementary Table S3. The BLAST results shown in Supplementary Table S3 demonstrate that for the strains analyzed by PCR they either possessed the alleles targeted by PCR, or that these alleles were absent. Interestingly, some strains contained either the toxin or the antitoxin component, but not both; and the prevalence and distribution of these TA homologs followed sequence type evolutionary lineages. Of the 234 strains analyzed for this experiment, toxin ESA_00804 (100%), antitoxin ESA_00803 (98%), and toxin ESA_01887 (99%) were the most conserved across the *C. sakazakii* strains of different sequence types. The only strains that contained all 22 TA homologs were *C. sakazakii* strains possessing ST1, but not every ST1 *C. sakazakii* contained all 22 TA pairs. 

**Figure 3:**
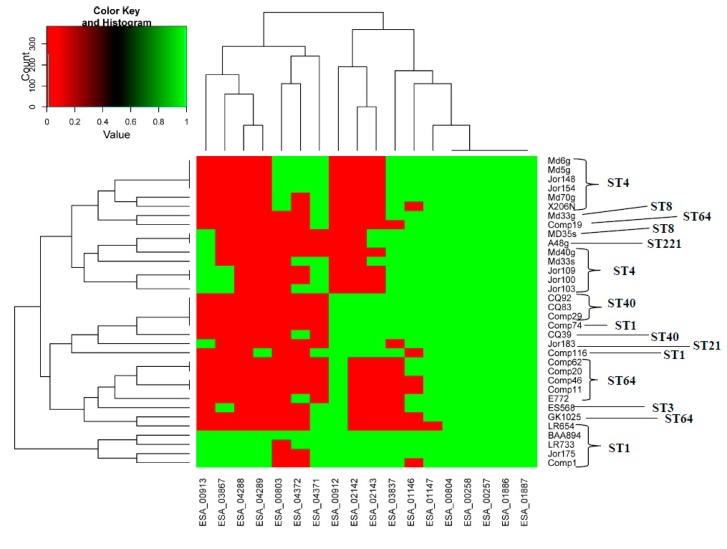
Thirty-four *C. sakazakii* genomes were analyzed by MA and BLAST against 18 Type II toxin-antitoxin sequences. Using a 90% BLAST homology cut off, the presence or absence of the TA gene in these strains was determined. This information was converted to a binary matrix 1 (Green) = present, 0 (Red) = absent. This was used in a python version 3 script that resulted in the development of a heat map. NOTE: Overlaying sequence type (ST) information onto the phylogeny created by this analysis showed that most of the strains clustered according to ST, as expected. BLAST analysis showed that not every *C. sakazakii* strain possessed all 18 Type II TA loci. This figure was meant only to serve as an example of what TAs are present.

Figure 3 shows a heat map with toxin and antitoxin homologs and the phylogeny among 34 *C. sakazakii* strains. This was created to give a visualization of the presence and absence of the TA homologs in these representative *C. sakazakii* strains. Manual overlaying of sequence type data obtained from the *Cronobacter* Pubmed MLST site (https://pubmlst.org/cronobacter/) again showed that most of the strains clustered according to sequence type. This figure also showed that not all toxin–antitoxin homologs were present in these representative *C. sakazakii* strains, which supported the previously reported BLAST, MA, and PCR results.

### 3.3. Acquisition of TAs Follow Different Evolutionary Incidences Which Draw a Parallel with Occurrence of ST Lineages

**Figure 4:**
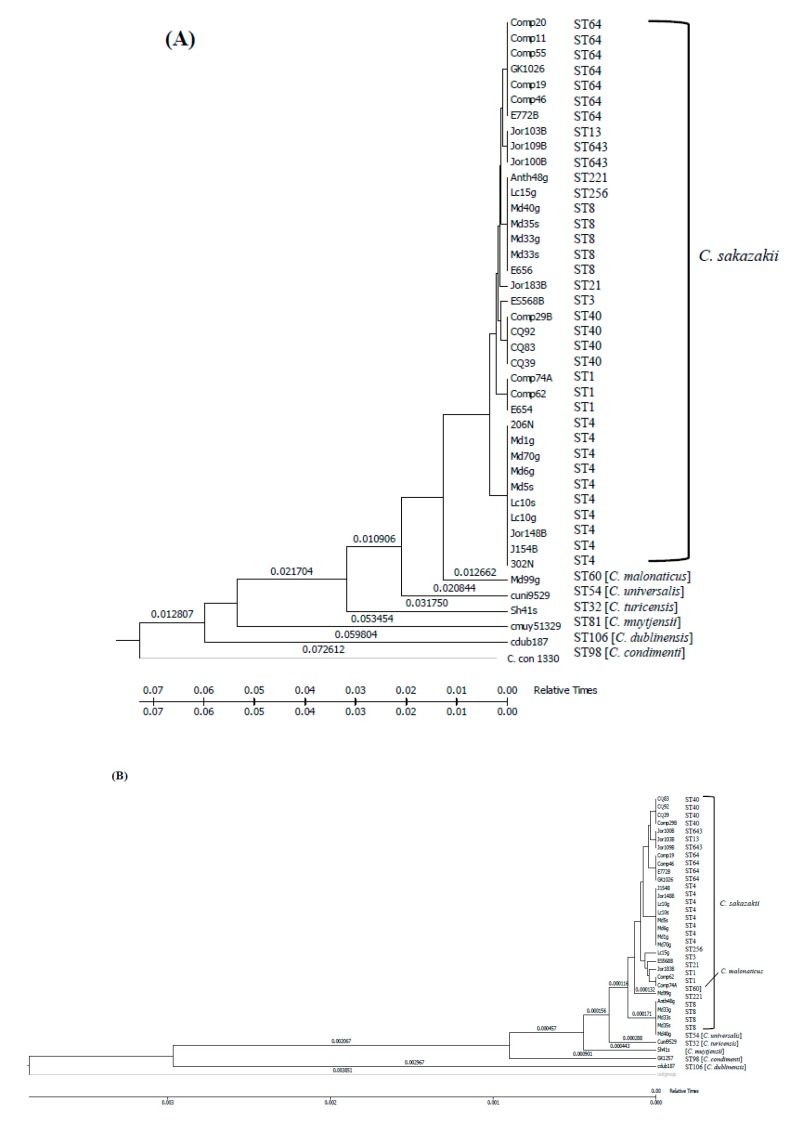
A timetree analysis of evolutionary relationship of *fic-like* (**A**) and *hipA* (**B**) genes among 42 *Cronobacter* species was inferred using the Reltime method described by Nei et al. [61] and Tamura et al. [62,63]. Estimates of branch lengths were inferred using the neighbor-joining method [55]. The analysis involved 42 nucleotide sequences of *fic-like* and 35 nucleotide sequences of *hipA* genes. There was a total of 603 positions for *fic-like* (**A**) and 1,320 positions for *hipA* (**B**) in the final dataset. Evolutionary analyses were conducted in MEGA7 [54]. All positions containing gaps and missing data were eliminated. The accession numbers for nucleotide sequences can be found in Table S1.

The molecular clock analysis shown in Figure 4 demonstrated that the *C. sakazakii* strains possibly originated from ancestral strain(s) that might have acquired TA genes at different evolutionary incidences; this draws a parallel with the occurrence of ST lineages. Recently Negrete et al. [60] reported similar evolutionary findings for efflux pumps possessed by *C. sakazakii*. We conjectured that the acquisition of these genes might have occurred either as independent evolutionary events through a horizontal gene transfer (such as through the attainment of plasmids, phage, or by transposition), or in certain situations, such as that of ST1, ST83, ST64, ST40, and ST4 strains, the TA genes could have been indiscriminately acquired through a robust microevolutionary selection process, also possibly involving horizontal gene transfer, some of which might have provided functional benefits. A more comprehensive analysis including candidates of closely related *Enterobacteriaceae* members might shed more light on how and when emergence of TA gene systems in *Cronobacter* occurred.

### 3.4. TA Association with Cronobacter Plasmids

It is well-known that the maintenance and stabilization of bacterial plasmids is controlled through the interplay of TAs genes by coupling them with both host cell division and plasmid propagation [4]. To better understand this relationship, we aligned three *C. sakazakii* virulence plasmids, pESA3, pSP291-1, and pCSK29544_1p that were reported by Franco et al. [64], Kucerova et al. [65] Power et al. [11], Yan et al. [47], and Moine et al. [66] and reproduced the alignment in CGView Server from the Stothard Research Group (http://stothard.afns.ualberta.ca/cgview_server/; last accessed 4 October 2019). The results (shown in Figure S1) illustrated that all three *C. sakazakii* virulence plasmids possessed and shared the *hipA* toxin gene and its antitoxin gene *xre* (annotated as a helix-turn-helix XRE-family transcriptional regulator). Interestingly, these C. *sakazakii* virulence plasmids also shared a common backbone consisting of an origin of replication gene (*repB*), iron ABC transporter (*eitCBAD*), and the only *Cronobacter* siderophore (*iucABCD/iutA*/*tonB* family siderophore/update gene cluster) with pCTU1 possessed by *C. turicensis* strain z3032^T^ (alias LMG 23827^T^), pCUNV1 possessed by *C. universalis* strain NCTC 9529^T^ (alias 797^T^), and *C. malonaticus* strain LMG 23826^T^ (alias CI825^T^) [64]. The presence of TA genes on *Cronobacter* plasmids needs further study using a larger sampling of isolates from different environmental and agricultural sources, to better understand their roles in persistence and pathogenicity.

### 3.5. TA Association with Cronobacter Phage

To understand the genomic relationship of TAs with that of prophage genomic regions that might be carried by *Cronobacter* strains, we first uploaded closed genome datasets for *C. sakazakii* strains BAA-894 (ST1, NCBI accession #: NC_009778) and ES15 (ST125, NCBI accession #: NC_017933) to the PHASTER web server and pipeline (https://phaster.ca/) for phage sequence identifications [67,68]. The PHASTER web server/pipeline identified two and three intact genomic regions in *C. sakazakii* strains BAA-894 and ES15, respectively. However, no TA genes were identified. Comparative sequence and phylogenetic analyses were then carried out using Geneious (http://www.geneious.com) and through annotation by BLAST it was observed that there was an intact *Salmonella* phage genomic region (identified as phage_118970_sal3, Refseq accession #:NC_031940) in the genomes of both BAA-894 and ES15 strains that contained a type II RelE/ParE family toxin-antitoxin system. In BAA-894, the *Salmonella* phage was only 40.2 kbp in size and in ES15, the phage was 42.8 kbp in size. Additionally, in BAA-894, the bicistronic TA operon was identified as a *parE-relE*/*parD-relB* type II TA system, where the antitoxin gene preceded the toxin gene and was located between base pair positions 98,5190–98,5426 (237 bp in size, encoding for a 79 amino acid protein) (originally annotated as a hypothetical protein). The toxin gene was located between 985,395–985,736 (342 bp in size, encoding for a 114 amino acid protein) and was identified as a RelE toxin. However, in ES15, the TA system was located from 1,346,920 to 1,369,940 bp and consisted of a bicistronic gene cluster where the antitoxin (location: 1,351,660–1,351,896, 237 bp in size, and encoding also for a 79 amino acid protein) was also originally annotated as a hypothetical protein, and was later annotated (through a wider BLAST analysis) as a *parD/relB* antitoxin that preceded the *parE/relE* toxin gene (location: 1,351,865–1,352,206, 342 bp in size, and encoding for a 114 amino acid protein). Other phages found in *C. sakazakii* strain ES15 included an intact *Cronobacter* phage (identified as phiES15, RefSeq accession #:NC_018454) that did not contain TAs within the phage genomic region. Although *Salmonella* phage_118970_sal3 was not as large as the P1 genome (93.6 kbp), the type II RelB/RelE-like toxin–antitoxins were found near the beginning of the phage genomic region (adjacent to a Holin encoding gene in both instances) where in P1 prophage, the type II Phd/Doc toxin–antitoxin gene cluster was found near the end of the genome region (adjacent to LP activation, packaging, and a repression of lytic function gene cluster) [7]. In contrast to P1, *Salmonella* phage118970_sal3 in the *Cronobacter* strains did not possess plasmid-like genes such as mobilization genes *parA*/*parB*, within its genome region, and the incompatibility class or origin of replication genes [7]. It might be speculated that the *Cronobacter* version of this *Salmonella* phage probably did not act as a plasmid like entity similar to that of prophage P1. Taken together, these results suggest that TAs associated with bacteriophage carried by *C. sakazakii* strains possess TAs that are both host-associated and phage-associated. 

## 4. Conclusions

On average, approximately 11 TAs such as *relE-xre*, COG5654-*xre*, *fic-phd*, *fic-yhfG,* and *hipA* loci with BLAST % nucleotide identity of >90% were found. A total of 109 TA homologs were confirmed to be present on the FDA *Cronobacter* microarray that represented the pan genome of the seven *Cronobacter* species. The TA allelic sequence divergence aligned along species taxa lines and within *C. sakazakii* aligned with ST lineages. WGS analysis of *fic*-like and a *hipA* orthologues of the seven species supported the phylogenetic relationship found by MA. Molecular clock analysis demonstrated that *C. sakazakii* strains possibly descended from an ancestral strain(s) that might have acquired TA genes at distinct evolutionary incidences that correlated with occurrence and evolution of ST lineages. We hypothesized that the possible acquisition of these genes could have occurred either as independent evolutionary events through horizontal gene transfer mechanisms (such as through the acquisition of plasmids or phage), or in certain situations TA genes could have been randomly acquired through a robust microevolutionary selective process, such as through transposition or plasmid acquisition, some of which might have provided functional advantages, e.g., the acquisition of plasmidborne virulence factor genes that provides the ability to survive in new econiches. Their identification is critical for a reliable prediction of gene function. Understanding the phylogenetic relatedness of TA genes among the *Cronobacter* spp. is essential for designing future studies to identify mechanisms used for survival and persistence in foods. These results demonstrate that in some species, TA homologs share a common phylogeny, while in other species, they follow species-specific phylogeny. Furthermore, these results add to the growing number of genomes of *Cronobacter* strains, many of which are from plant–host origins. Availability of genomic information from these strains would provide a better understanding of the genetic features linked to plant association and expand insights into the possible evolutionary history of this important foodborne pathogen.

## Figures and Tables

**Table 1:** Strain information of *Cronobacter, Siccibacter,* and *Franconibacter* isolates used for the phylogenetic microarray analysis in this study.

Strain Name	Species ID	ST ^a^, CC ^b^	Source	Country	NCBI Accession No.
Comp1	*C. sakazakii*	ST1, CC1	Environment, dairy powder manufacturing facility	USA	WAGE00000000
Comp11	*C. sakazakii*	ST64, CC64	Environment, dairy powder manufacturing facility	USA	NHQL01000000
Comp19	*C. sakazakii*	ST64, CC64	Environment, dairy powder manufacturing facility	USA	NHQM00000000
Comp20	*C. sakazakii*	ST64, CC64	Environment, dairy powder manufacturing facility	USA	NEXY00000000
Comp29	*C. sakazakii*	ST40, CC40	Environment, dairy powder manufacturing facility	USA	Not available
Comp46	*C. sakazakii*	ST64, CC64	Environment, dairy powder manufacturing facility	USA	NEYA00000000
Comp62	*C. sakazakii*	ST1, CC1	Environment, dairy powder manufacturing facility	USA	WAGM00000000
Comp74	*C. sakazakii*	ST1, CC1	Environment, dairy powder manufacturing facility	USA	Not available
Comp116	*C. sakazakii*	ST1, CC1	Environment, dairy powder manufacturing facility	USA	WAGR00000000
Jor175	*C. sakazakii*	ST1, CC1	Food, spices	Jordan	NITO00000000
LR733	*C. sakazakii*	ST1, CC1	Food, organic flour	USA	PTOU00000000
CQ32	*C. sakazakii*	ST1, CC1	Environment, powdered infant formula facility	Ireland	WAGV00000000
BAA-894	*C. sakazakii*	ST1, CC1	Food, powdered infant formula	USA	CP000783
Jor100	*C. sakazakii*	ST643, CC13	Food, semolina	Jordan	NITS01000000
Jor103	*C. sakazakii*	ST643, CC13	Food, spices	Jordan	NITR00000000
Jor109	*C. sakazakii*	ST643, CC13	Food, grapes	Jordan	NITQ00000000
Jor148	*C. sakazakii*	ST4, CC4	Food, spices	Jordan	PVCF00000000
Jor154	*C. sakazakii*	ST4, CC4	Food, spices	Jordan	NITP00000000
Jor183	*C. sakazakii*	ST21, CC21	Food, spices	Jordan	NITN00000000
CQ39	*C. sakazakii*	ST40, CC40	Environment, powdered infant formula facility	Ireland	WAGW00000000
CQ83	*C. sakazakii*	ST40, CC40	Environment, powdered infant formula facility	Ireland	Not available
CQ92	*C. sakazakii*	ST40, CC40	Environment, powdered infant formula facility	Ireland	Not available
GK1025	*C. sakazakii*	ST64, CC64	Environment, powdered infant formula facility	Germany	MCOE00000000
Md33s	*C. sakazakii*	ST8, CC8	Fly, *Musca domestica*, surface	USA	MRXC00000000
Md33g	*C. sakazakii*	ST8, CC8	Fly, *Musca domestica*, gut	USA	MSAI00000000
Md35s	*C. sakazakii*	ST8, CC8	Fly, *Musca domestica*, surface	USA	MRXD00000000
Md40g	*C. sakazakii*	ST8, CC8	Fly, *Musca domestica*, gut	USA	MRXE00000000
Anth48g	*C. sakazakii*	ST221	Fly, *Anthomyiidae* spp., gut	USA	MRXF00000000
Md5s	*C. sakazakii*	ST4, CC4	Fly, *Musca domestica*, surface	USA	MRWZ00000000
Md6g	*C. sakazakii*	ST4, CC4	Fly, *Musca domestica*, gut	USA	MRXB00000000
Md70g	*C. sakazakii*	ST4, CC4	Fly, *Musca domestica*, gut	USA	MRXG00000000
Md1g	*C. sakazakii*	ST4, CC4	Fly, *Musca domestica*, gut	USA	MSAH00000000
206N	*C. sakazakii*	ST4, CC4	Clinical	Ireland	WAEU00000000
ES568	*C. sakazakii*	ST3, CC3	Environment, powdered infant formula facility	Switzerland	Not available
E654	*C. sakazakii*	ST1, CC1	Clinical	Ireland	NCWF00000000
E772	*C. sakazakii*	ST64, CC64	Food, milk powder	France	NHQS00000000
GK1257	*C. muytjensii*	ST546	Environment, powdered infant formula facility	Germany	WAGD00000000
Md99g	*C. malonaticus*	ST60	Fly, *Musca domestica*, gut	USA	MSAF00000000
Md1sN	*C. turicensis*	ST519	Fly, *Musca domestica*, gut	USA	VOEL00000000
Sh41s	*C. turicensis*	ST569	Fly, *Sarcophaga haemorrhoidalis*, surface	USA	MSAG01000000
Sh41g	*C. turicensis*	ST569	Fly, *Sarcophaga haemorrhoidalis*, gut	USA	MRZS00000000
NCTC 9529^T^ (797)	*C. universalis*	ST54	Environment, water	UK	NZ_CP012257
51329^T^	*C. muytjensii*	ST81	Unknown	USA	NZ_CP012268
187 (LMG23823^T^)	*C. dublinensis*	ST106	Environment, powdered infant formula facility	Ireland	NZ_CP012266
1330 (LMG 26250^T^)	*C. condimenti*	ST98	Food, spiced sausage	Slovakia	NZ_CP012264
464 (LMG23825^T^)	*C. dublinensis*	ST79	Environment, milk powder production facility	Zimbabwe	AJKX00000000
CI825 (LMG23826^T^)	*C. malonaticus*	ST7	Clinical, breast abscess	USA	NZ_CP013940
508 (LMG 23730^T^)	*Siccibacter turicensis*	N/A ^c^	Food, fruit powder	Switzerland	AWFZ01000000
601 (LMG 24057^T^)	*Franconibacter pulveris*	N/A	Food, fruit powder	Switzerland	AXSY00000000
1160 (LMG 24058^T^)	*Franconibacter pulveris*	N/A	Food, fruit powder	Switzerland	AXSZ00000000
513 (LMG 23732^t^)	*Franconibacter helveticus*	N/A	Food, fruit powder	Switzerland	AXDK00000000

^a^ Sequence type (ST) was determined by uploading genome assemblies to https://pubmlst.org/cronobacter (last accessed 30 September 2019). ^b^ CC, clonal complex. ^c^ N/A refers to a “not applicable”.

**Table 2:** Description of the PCR primers, amplicon sizes, and PCR reaction parameters ^#^ targeting five common *C. sakazakii* toxin genes used in this study.

Primer Target ^a^	Forward and Reverse Primer	Sequence (5′-3′)	Amplicon Size
ESA00258	00258F5	CGA GAC CGT TAA AGC GCA AT	211 bp
	00258R3	CCC CTG GTA TAC GGT CAG GT	
ESA00804	00804F10	TGG AGA TCA GAT GGA CGA AGC	251 bp
	00804R9	TGT GGT TGT CGT TCT GCG TT	
ESA01887	01887F2	TCA GGC ATA AAG GCC TGC AA	239 bp
	01887R7	AAA GAC ATC GCC ATC CCG AA	
ESA03838	03838F3	AAT TTT TCA TCC GGT CGC GG	301 bp
	03838R4	ATG GCT GAG CTC CTC CAA TC	
ESA04372	04372F7	GCG CGA CCC TTA TTT CTG GT	538 bp
	04372R1	TTT TCT CAA GCG GTG CCA GA	

^#^ PCR reaction parameters for all primers consisted of first activating the GoTaq Hotstart DNA polymerase in the GoTaq Green master mix (Promega Corp., Madison, WI), by using a 3-min incubation step at 95 °C, followed by 30 cycles of denaturation at 94 °C for 30s, annealing at 57 °C and amplicon extension at 72 °C for 40 s. For each reaction, a final extension step of 5 min at 72 °C was used. All PCR mixtures were prepared using a 25-µL reaction mixture with 1 unit of GoTaq Hotstart DNA polymerase, 1.5 mM MgCl_2_, and 200 µM each of deoxynucleoside triphosphate. Primers were added at 1 µM each, and 1 µL of the DNA sample (approximately 90 ng DNA/25-µL reaction mixture). ^a^ Toxin primer targets and representative toxin–antitoxin families are—ESA_00258 (parDE toxin–antitoxin family), ESA_00804 (relBE family), ESA_01887 (relBE family), ESA_03838 (relBE family), and ESA_04273 (YhfG-Fic family).

**Table 3:** A summary table as an example of the toxin diversity that is among the representative *C. sakazakii* strains obtained from environmental samples of dairy powder manufacturing facilities that had at least one negative PCR result.

Strain	Toxin PCR Patterns Observed among 22 *C. sakazakii* Strains				
	ESA_00258	ESA_00804	ESA_01887	ESA_03838	ESA_04372
Comp 11	+	+	+	−	+
Comp 13	+	+	+	−	+
Comp 14	+	+	+	−	+
Comp 15	+	+	+	−	+
Comp 18	+	+	+	−	+
Comp 19	+	+	+	−	+
Comp 20	+	+	+	−	+
Comp 26	+	−	+	+	+
Comp 28	−	+	−	−	−
Comp 42	+	+	+	−	+
Comp 45	+	+	+	−	+
Comp 46	+	+	+	−	+
Comp 48	+	+	+	−	+
Comp 49	−	+	+	−	+
Comp 52	+	+	+	−	+
Comp 53	+	+	+	−	+
Comp 54	+	+	+	−	+
Comp 55	−	+	−	−	+
Comp 57	+	+	+	−	+
Comp 58	+	+	+	−	+
Comp 59	+	+	+	−	+
Comp 60	+	+	+	−	+

**Table 4:** Summary table of percent distribution for each of the twenty-two TA genes in the 234 *C. sakazakii* strains analyzed using the local database. TA FASTA sequences downloaded from TADB were used as the query in the local database blasting against the *C. sakazakii* genomes sequenced by WGS.

Toxin (T) or Antitoxin (A)	Toxin Gene	NCBI Annotations	Presence in *C. saks* (%)
A	ESA_00257	RelB protein (antitoxin to RelE)	83
T	ESA_00258	RelE antibacterial toxin protein	88
A	ESA_00803	transcriptional regulator2C XRE family	100
T	ESA_00804	hypothetical protein	100
T	ESA_00912	FIG00554131: hypothetical protein	13
A	ESA_00913	FIG00553297: hypothetical protein	38
T	ESA_01146	hypothetical protein	18
A	ESA_01147	Putative transcriptional regulator	94
A	ESA_01886	HigA protein (antitoxin to HigB)	37
T	ESA_01887	HigB toxin protein	100
A	ESA_02142	HigA protein (antitoxin to HigB)	75
T	ESA_02143	HigB toxin protein	21
A	ESA_03837	FIG00554128: hypothetical protein	76
T	ESA_03838	FIG00554128: hypothetical protein	77
A	ESA_03866	Putative merR family bacterial regulatory protein	29
T	ESA_03867	FIG00642734: hypothetical protein	13
A	ESA_04288	Prevent host death protein2C Phd antitoxin	14
T	ESA_04289	Death on curing protein2C Doc toxin	13
A	ESA_04371	FIG00553654: hypothetical protein	58
T	ESA_04372	Cell filamentation protein fic	91
T	ESA3p05543	hypothetical_protein	14
A	ESA3p05544	hypothetical_protein	23

## Supplementary Materials

The following are available online at https://www.mdpi.com/2076-2607/7/11/554/s1, Figure S1: Alignment of three C. sakazakii virulence plasmids, pESA3, pSP291-1, and pCSK29544_1p produced by using CGView Server from the Stothard Research Group (http://stothard.afns.ualberta.ca/cgview_server/; last accessed 25 June 2018) showing the alignment of hipA and its antitoxin xre among the three *C. sakazakii* virulence plasmids The software uses BLAST analysis to illustrate homology among the toxin homologs as well as presence of conserved or the absence of missing homologs. Two circular plasmids—pSP291-1 (NC_020263) and pCSK29544_1p (NZ_CP011048) – were compared against reference plasmid, pESA3 (NC_009780). *hipA* family toxin and a Helix-Turn-Helix (HTH-XRE) domain antitoxin (originally identified as XRE-family transcriptional regulator) are shown; Table S1: *Cronobacter* strains including the *C. sakazakii* strain names, sequence type (ST) assignment, source, country, and NCBI accession numbers used in the BLAST analysis experiment described in Supplemental Table S3; Table S2: One hundred and nine toxin-antitoxin probe, probeset, probe species ID that the probe sequence came from, % similarity to BAA-894 allele, NCBI protein accession #, NCBI annotations, loci, and genome region (GR) which are located on the FDA Cronobacter microarray as described by Tall et al. [53]. Fifty *Cronobacter* strains were evaluated using these homologs and the results were used to develop the phylogenetic tree shown in Figure 1, Table S3: Summary of local BLAST results using the 22 toxin-antitoxin loci against 234 *C. sakazakii* strains to identify which TA allele is present in each strain. Strains were identified as *C. sakazakii* by were identified using species-specific (*rpoB* and *cgcA*) PCR assays [47,48,49]. Multi-locus sequence typing (MLST) analysis of the strains was carried out following the protocol described by Joseph et al. [50].
