# Supplementary material for: Prevalence, Distribution, and Phylogeny of Type Two Toxin-Antitoxin Genes Possessed by Cronobacter Species where C. sakazakii Homologs Follow Sequence Type Lineages
Source: Microorganisms. 2019 Nov 12;7(11):554. doi: 10.3390/microorganisms7110554 (PMC6920972; doi:10.3390/microorganisms7110554)
Supplement: Supplementary file 1 [file microorganisms-07-00554-s001.zip › Supplement Tables 1-3/Supplement Table 1_10.3.2019.docx]

**Supplemental Table 1.** *Cronobacter* *sakazakii* strain names, sequence type (ST) assignment, source, country, and NCBI accession numbers used in the BLAST analysis experiment described in **Supplemental Table 3**.

| Strain Name | ST^a^, CC^b^ | Source | Country | NCBI Accession or SRA no. |
| --- | --- | --- | --- | --- |
| Comp1 | ST1 | Environment, dairy powder manufacturing facility | USA | WAGE00000000 |
| Comp4 | ST31 | Environment, dairy powder manufacturing facility | USA | WAGF00000000 |
| Comp9 | ST4 | Environment, dairy powder manufacturing facility | USA | WAGG00000000 |
| Comp10 | ST40 | Environment, dairy powder manufacturing facility | USA | WAGH00000000 |
| Comp11 | ST64, CC64 | Environment, dairy powder manufacturing facility | USA | NHQL01000000 |
| Comp13 | ST83 | Environment, dairy powder manufacturing facility | USA | WAGI00000000 |
| Comp15 | ST83 | Environment, dairy powder manufacturing facility | USA | NCTU00000000 |
| Comp19 | ST64, CC64 | Environment, dairy powder manufacturing facility | USA | NHQM00000000 |
| Comp20 | ST64, CC64 | Environment, dairy powder manufacturing facility | USA | NEXY00000000 |
| Comp33 | ST4 | Environment, dairy powder manufacturing facility | USA | WAGJ00000000 |
| Comp36 | ST4 | Environment, dairy powder manufacturing facility | USA | WAGK00000000 |
| Comp45 | ST64 | Environment, dairy powder manufacturing facility | USA | NEXZ00000000 |
| Comp46 | ST64, CC64 | Environment, dairy powder manufacturing facility | USA | NEYA00000000 |
| Comp47 | ST83 | Environment, dairy powder manufacturing facility | USA | NCTT00000000 |
| Comp49 | ST64 | Environment, dairy powder manufacturing facility | USA | NEYB00000000 |
| Comp53 | ST64 | Environment, dairy powder manufacturing facility | USA | NEYC00000000 |
| Comp54 | ST64 | Environment, dairy powder manufacturing facility | USA | NEYD00000000 |
| Comp57 | ST64 | Environment, dairy powder manufacturing facility | USA | NEYE00000000 |
| Comp59 | ST64 | Environment, dairy powder manufacturing facility | USA | NEYF00000000 |
| Comp60 | ST83 | Environment, dairy powder manufacturing facility | USA | WAGL00000000 |
| Comp62 | ST1, CC1 | Environment, dairy powder manufacturing facility | USA | WAGM00000000 |
| Comp67 | ST198, CC52 | Environment, dairy powder manufacturing facility | USA | WAGN00000000 |
| Comp84 | ST99 | Environment, dairy powder manufacturing facility | USA | PVSQ00000000 |
| Comp88 | ST226, CC8 | Environment, dairy powder manufacturing facility | USA | PVBY00000000 |
| Comp95 | ST1 | Environment, dairy powder manufacturing facility | USA | WAGO00000000 |
| Comp107 | ST1 | Environment, dairy powder manufacturing facility | USA | WAGP00000000 |
| Comp108 | ST1 | Environment, dairy powder manufacturing facility | USA | WAGQ00000000 |
| Comp116 | ST1 | Environment, dairy powder manufacturing facility | USA | WAGR00000000 |
| Comp128 | ST198, CC52 | Environment, dairy powder manufacturing facility | USA | WAGS00000000 |
| CQ14 | ST4 | Environment, PIF^c^ facility | Ireland | WAGT00000000 |
| CQ19 | ST4 | Environment, PIF facility | Ireland | WAGU00000000 |
| CQ32 | ST1 | Environment, PIF facility | Ireland | WAGV00000000 |
| GK1160 | ST1 | Environment, PIF facility | Germany | WAEY00000000 |
| GK1259 | ST1 | Environment, PIF facility | Germany | WAEZ00000000 |
| GK1260 | ST1 | Environment, PIF facility | Germany | WAFA00000000 |
| GK1261 | ST1 | Environment, PIF facility | Germany | WAFB00000000 |
| GK793 | ST1 | Environment, PIF facility | Germany | WAFC00000000 |
| GK960 | ST1 | Environment, PIF facility | Germany | WAFD00000000 |
| GK1032 | ST1 | Environment, PIF facility | Germany | WAFE00000000 |
| GK1033 | ST4 | Environment, PIF facility | Germany | WAFF00000000 |
| GK1036 | ST4 | Environment, PIF facility | Germany | WAFG00000000 |
| GK1154 | ST4 | Environment, PIF facility | Germany | WAFH00000000 |
| GK1155 | ST4 | Environment, PIF facility | Germany | WAFI00000000 |
| GK1156 | ST4 | Environment, PIF facility | Germany | WAFJ00000000 |
| GK1157 | ST4 | Environment, PIF facility | Germany | WAFK00000000 |
| GK1158 | ST4 | Environment, PIF facility | Germany | WAFL00000000 |
| GK1159 | ST4 | Environment, PIF facility | Germany | WAFM00000000 |
| GK1263 | ST4 | Environment, PIF facility | Germany | WAFN00000000 |
| GK1264 | ST4 | Environment, PIF facility | Germany | WAFO00000000 |
| GK798 | ST4 | Environment, PIF facility | Germany | WAFP00000000 |
| GK8011 | ST4 | Environment, PIF facility | Germany | WAFQ00000000 |
| GK8012 | ST4 | Environment, PIF facility | Germany | WAFR00000000 |
| GK950 | ST4 | Environment, PIF facility | Germany | WAFS00000000 |
| GK951 | ST4 | Environment, PIF facility | Germany | WAFT00000000 |
| GK952 | ST4 | Environment, PIF facility | Germany | WAFU00000000 |
| GK953 | ST4 | Environment, PIF facility | Germany | WAFV00000000 |
| GK954 | ST4 | Environment, PIF facility | Germany | WAFW00000000 |
| GK956 | ST4 | Environment, PIF facility | Germany | WAFX00000000 |
| GK957 | ST4 | Environment, PIF facility | Germany | WAFY00000000 |
| GK958 | ST4 | Environment, PIF facility | Germany | WAFZ00000000 |
| GK959 | ST4 | Environment, PIF facility | Germany | WAGA00000000 |
| GK963 | ST4 | Environment, PIF facility | Germany | WAGB00000000 |
| GK964 | ST4 | Environment, PIF facility | Germany | WAGC00000000 |
| GK1025 | ST64 | Environment, PIF facility | Germany | MCOE00000000 |
| GK1027 | ST64 | Environment, PIF facility | Germany | NHQN00000000 |
| GK1029 | ST64 | Environment, PIF facility | Germany | NHQO00000000 |
| GK1030 | ST64 | Environment, PIF facility | Germany | NHQP00000000 |
| GK1034 | ST64 | Environment, PIF facility | Germany | NHQQ00000000 |
| GK1035 | ST64 | Environment, PIF facility | Germany | NHQR00000000 |
| GK1326 | ST64 | Environment, PIF facility | Germany | NEYH00000000 |
| GK1026 | ST64, CC64 | Environment, PIF facility | Germany | NEYG00000000 |
| GK1262 | ST83 | Environment, PIF facility | Germany | NCTO00000000 |
| GK794 | ST83 | Environment, PIF facility | Germany | NCTR00000000 |
| GK799 | ST83 | Environment, PIF facility | Germany | NCTQ00000000 |
| GK800 | ST83 | Environment, PIF facility | Germany | NCTP00000000 |
| GK792.3 | ST83, CC83 | Environment, PIF facility | Germany | NCTS00000000 |
| LR626 | ST1 | Food, rice flour | USA | PVCR00000000 |
| LR704 | ST1 | Food, flour | USA | QHGX00000000 |
| LR707 | ST1 | Food, organic flour | USA | PTOZ00000000 |
| LR708 | ST1 | Food, organic flour | USA | PTOY00000000 |
| LR733 | ST1 | Food, organic flour | USA | PTOU00000000 |
| LR735 | ST1 | Food, whey protein | USA | PVDH00000000 |
| LR652 | ST3 | Food, baby's cereal with bananas | USA | WARI00000000 |
| LR631 | ST4 | Food, instant oatmeal with strawberries and cream | USA | PTPF00000000 |
| LR691 | ST4 | Food, soy protein | USA | WARJ00000000 |
| LR705 | ST4 | Food, organic soy powder | USA | QHGW00000000 |
| LR706 | ST4 | Food, organic soy | USA | PVCU00000000 |
| LR702 | ST21 | Food, PIF | USA | PVDG00000000 |
| LR703 | ST22 | Food, flour | USA | PVCT00000000 |
| LR635 | ST148, CC16 | Food, dried cut carrots | USA | PTPC00000000 |
| LR712 | 6 alleles matches^d^ | Food, organic soy | USA | PVCW00000000 |
| Jor175 | ST1 | Food, spices | Jordan | NITO00000000 |
| Jor146 | ST3 | Food, liquorice | Jordan | PVMV00000000 |
| Jor96 | ST4 | Food, fennel, spice | Jordan | PVCE00000000 |
| Jor44 | ST8, CC8 | Food, spices | Jordan | PVCC00000000 |
| Jor20 | ST226, CC8 | Food, spices | Jordan | PVCA00000000 |
| Jor22 | ST226, CC8 | Food, chamomile | Jordan | PVCB00000000 |
| Jor93 | ST13 | Food, spices | Jordan | PVCD00000000 |
| Jor204 | ST22 | Food, liquorice | Jordan | QHGV00000000 |
| Jor172 | ST64 | Food, Spices | Jordan | NCWD00000000 |
| Jor100 | ST643, CC13 | Food, semolina | Jordan | NITS01000000 |
| Jor103 | ST643, CC13 | Food, spices | Jordan | NITR00000000 |
| Jor109 | ST643, CC13 | Food, grapes | Jordan | NITQ00000000 |
| Jor148 | ST4, CC4 | Food, spices | Jordan | PVCF00000000 |
| Jor154 | ST4, CC4 | Food, spices | Jordan | NITP00000000 |
| Jor183 | ST21, CC21 | Food, spices | Jordan | NITN00000000 |
| Md5g | ST4 | Fly, *Musca domestica*, gut | USA | MRXA00000000 |
| Md27gN | ST93 | Fly, *Musca domestica*, gut | USA | VOEK00000000 |
| Md33s | ST8 | Fly, *Musca domestica*, surface | USA | MRXC00000000 |
| Md33g | ST8 | Fly, *Musca domestica*, gut | USA | MSAI00000000 |
| Md35s | ST8 | Fly, *Musca domestica*, surface | USA | MRXD00000000 |
| Md40g | ST8 | Fly, *Musca domestica*, gut | USA | MRXE00000000 |
| Ls15g | ST256 | Fly, *Lucilla sericata*, gut | USA | NDXF00000000 |
| Anth48g | ST221 | Fly, *Anthomyiidae* spp., gut | USA | MRXF00000000 |
| Lc10s | ST4 | Fly, *Lucilla cuorina*, surface | USA | NDXD00000000 |
| Lc10g | ST4 | Fly, *Lucilla cuorina*, gut | USA | NDXE00000000 |
| Md5s | ST4 | Fly, *Musca domestica*, surface | USA | MRWZ00000000 |
| Md6g | ST4 | Fly, *Musca domestica*, gut | USA | MRXB00000000 |
| Md70g | ST4 | Fly, *Musca domestica*, gut | USA | MRXG00000000 |
| Md1g | ST4 | Fly, *Musca domestica*, gut | USA | MSAH00000000 |
| KW13 | ST13 | Food, dried garlic | Republic of Korea | NITD00000000 |
| KW3 | ST40 | Food, dried hot pepper | Republic of Korea | NITH00000000 |
| KW1 | ST93 | Food, barley | Republic of Korea | NITI00000000 |
| KW9 | ST143 | Food, sorghum | Republic of Korea | NITF00000000 |
| KW18 | ST156, CC21 | Food, mushroom | Republic of Korea | NITC00000000 |
| KW4 | ST73 | Food, dried Seaweed | Republic of Korea | NITG00000000 |
| KW2 | ST222 | Food, dried filefish | Republic of Korea | WARK00000000 |
| KW11 | ST642 | Food, black bean | Republic of Korea | NITE00000000 |
| KW10 | 7 alleles matches^e^ | Food, powdered pine needles | Republic of Korea | WARL00000000 |
| ES33 | ST83 | Clinical | Israel | WAEP00000000 |
| E657 | ST1 | Clinical | Ireland | WAEQ00000000 |
| E899 | ST4 | Clinical | USA | AFMO00000000 |
| ES32 | ST4 | Clinical | Israel | WAER00000000 |
| ES34 | ST4 | Clinical | Israel | WAES00000000 |
| E837 | ST13 | Clinical | France | WAET00000000 |
| E654 | ST1, CC1 | Clinical | Ireland | NCWF00000000 |
| E656 | ST8, CC8 | Clinical | Ireland | WARO00000000 |
| E772 | ST64, CC64 | Food, milk powder | France | NHQS00000000 |
| 206N | ST4, CC4 | Clinical | Ireland | WAEU00000000 |
| 302N | ST4, CC4 | Clinical | Ireland | WAEV00000000 |
| 254N | ST1 | Clinical | Ireland | NCWG00000000 |
| 305N | ST1 | Clinical | Ireland | WAEW00000000 |
| 3-21 | ST1 | Food, nuts | Republic of Korea | NITK00000000 |
| NM1241 | ST1 | Clinical | USA | NCWE00000000 |
| C019565 | ST1, CC1 | Environmental swab | USA | SRS726897 |
| BAA-894 | ST1, CC1 | Food, infant formula | USA | NC_009778 |
| C019574 | ST13, CC13 | Environmental swab | USA | SRS726948 |
| 5-20G | ST17, CC17 | Food, nuts | Republic of Korea | NITL00000000 |
| 5-21G | ST17, CC17 | Food, nuts | Republic of Korea | NIXM00000000 |
| 701753 | ST31, CC31 | Environment, Meade Johnson plant near a tote of vitamin premixes | USA | SRS3465726 |
| C019575 | ST31, CC31 | Environmental swab | USA | SRS726895 |
| C019573 | ST31, CC31 | Environmental swab | USA | SRS726917 |
| C019576 | ST40, CC40 | Environmental swab | USA | SRS726939 |
| HPB5174 | ST40, CC40 | Environmental swab, food processing facility | Ireland | JNBN00000000 |
| 680 | ST8 | Clinical | USA | CALG00000000 |
| 3128-77 | ST50 | Clinical | USA | SRS3436344 |
| H1691AC | ST64 | Environment, PIF facility | Switzerland | NHTV00000000 |
| 1121-73 | ST64 | Clinical, bronchial wash | USA | MCOD00000000 |
| cro2819A3 | ST64, CC64 | Food, mushroom | China | MBSC00000000 |
| 701 | ST81 | Unknown | USA | CALE01000000 |
| A31 | ST83 | Environment, PIF facility | Switzerland | MRXT00000000 |
| H1191 | ST83 | Environment, PIF facility | Switzerland | MRXQ00000000 |
| H2397 | ST83 | Environment, PIF facility | Switzerland | MRXV00000000 |
| H2399 | ST83 | Environment, PIF facility | Switzerland | MRXI00000000 |
| H322 | ST83 | Environment, PIF facility | Switzerland | MRXM00000000 |
| ES15 | ST125 | Food, ground wholegrains | Republic of Korea | CP003312 |
| 1-15 | ST136 | Food, nuts | Republic of Korea | NITJ00000000 |
| GP1999 | ST145 | *Rhizospere* of tomato plant | USA | NHTW00000000 |
| C022615 | ST184, CC142 | Environmental swab | USA | SRS722936 |
| C022618 | ST184, CC142 | Environmental sample | USA | SRS722933 |
| C019572 | ST198, CC52 | Environmental swab | USA | SRS726898 |
| SRR1614319 | ST233 | Environmental sample | USA | SRS722937 |
| E602 | ST3 | Clinical | Ireland | WARM00000000 |
| 33SS8 | ST4 | Environment, PIF facility | Switzerland | MRXR00000000 |
| 1242 | ST4 | Clinical, brain | USA | SRS3436352 |
| Cr14 | ST4 | Clinical, CSF^d^ | USA | NDXX00000000 |
| 207N | ST4 | Clinical | Ireland | SRS3436334 |
| 208N | ST4 | Clinical | Ireland | SRS3436335 |
| 2148 | ST4 | Clinical, blood | USA | SRS3436353 |
| 2150 | ST4 | Clinical, tracheal aspirate | USA | SRS3436356 |
| 2151 | ST4 | Clinical, CSF | USA | NCWM00000000 |
| 2152 | ST4 | Clinical, CSF | USA | SRS3436357 |
| 2154 | ST4 | Clinical, CSF | USA | SRS3436367 |
| 2155 | ST4 | Clinical, CSF | USA | SRS3436365 |
| 2156 | ST4 | Clinical, blood | USA | SRS3436366 |
| 2157 | ST4 | Clinical, stool | USA | SRS3436364 |
| 228N | ST4 | Clinical | Ireland | SRS3436336 |
| 229N | ST4 | Clinical | Ireland | SRS3436337 |
| 255N | ST4 | Clinical | Ireland | SRS3436328 |
| 303N | ST4 | Clinical | Ireland | SRS3436329 |
| 304N | ST4 | Clinical | Ireland | SRS3436349 |
| 32B1C | ST4 | Environment, PIF facility | Switzerland | MRXS00000000 |
| 306N | ST4 | Clinical | Ireland | SRS3436351 |
| 35ss5 | ST4 | Environment, PIF facility | Switzerland | MRXU00000000 |
| E899 | ST4 | Clinical | USA | AFMO00000000 |
| Cr3 | ST4 | Clinical, brain exudate | USA | SRS3465747 |
| Cr4 | ST4 | Environment, baby pacifer | USA | NCWP00000000 |
| Cr5 | ST4 | Clinical, rectal swab, stool | USA | NCWN00000000 |
| Cr6 | ST4 | Food, PIF | USA | SRS3465721 |
| Cr7 | ST4 | Clinical, CSF | USA | SRS3465720 |
| Cr10 | ST4 | Food, PIF | USA | NCWJ00000000 |
| Cr13 | ST4 | Food, water | USA | NCWI00000000 |
| H2395 | ST4 | Environment, PIF facility | Switzerland | MRXL00000000 |
| H269 | ST4 | PIF control | Switzerland | MRXP00000000 |
| H482 | ST4 | PIF control | Switzerland | MRXK00000000 |
| H951 | ST4 | PIF control | Switzerland | MRXO00000000 |
| SK1180 | ST4 | Environment, PIF facility | Switzerland | MRXH00000000 |
| SP291 | ST4 | Environment, PIF facility | Ireland | NC_020260 |
| SP4 | ST4 | Environment, PIF facility | Switzerland | MRXW00000000 |
| Cr11 | ST4, CC4 | Food, reconstituted PIF | USA | SRS3465724 |
| A31815 | ST4, CC4 | PIF control | Switzerland | MRXN00000000 |
| NM1240 | ST4, CC4 | Clinical, CSF | USA | JZDO00000000 |
| S1765 | ST4, CC4 | Food, PIF product isolate | Switzerland | MRXJ00000000 |
| 20112103 | ST4, CC4 | Clinical, CSF | USA | SRS3465737 |
| SRR1614320 | ST4, CC4 | Environmental swab, taken from whey drying equipment | USA | SRS722938 |
| 8272x | ST4, CC4 | Food, milk powder | UK | AWFW00000000 |
| 8399x | ST4, CC4 | Clinical, CSF | Israel | AWSP00000000 |
| C019579 | ST4, CC4 | Environmental swab | USA | SRS726919 |
| 607A | ST218, CC4 | Clinical | USA | SRS3436361 |
| 29544 | ST8 | Clinical, child's throat | USA | NZ_CP011047 |
| 696 | ST8 | Clinical | France | JOLW00000000 |
| Cr8 | ST8 | Food, nursery water | USA | NCWC00000000 |
| Cr9 | ST8 | Food, nursery water | USA | SRS3465722 |
| 5-17G | ST226, CC8 | Food, nuts | Republic of Korea | NITM00000000 |
| 40777 | ST8, CC8 | Clinical, sputum | USA | SRS3465754 |
| 2011180503 | ST8 | Food, opened PIF isolate | USA | WBSH00000000 |
| Cr15 | ST8, CC8 | Clinical, patient stool isolate | USA | SRS3465725 |
| CDC105977 | ST8, CC8 | Clinical | USA | WARN00000000 |
| NBRC102416 | ST8, CC8 | Clinical | USA | BAWU00000000 |
| ES35 | ST8, CC8 | Clinical | Israel | AJLC00000000 |
| E764 | ST12 | Clinical | Czech Republic | AJLA00000000 |
| CDC936375 | ST12 | Unknown | USA | WBSD00000000 |
| 496371 | ST1, CC1 | Clinical, stool | USA | WAEX00000000 |
| Cr2 | 6 alleles matches^f^ | Clinical, blood | USA | NCWO00000000 |

^a^ Sequence type (ST) was determined by uploading genome assemblies to https://pubmlst.org/cronobacter (last accessed 10/1/2019).

^b^ CC, clonal complex.

^c^ PIF, powdered infant formula.

^d^ 6 alleles matches are found in atpD 5, glnS 3, gltB 3, gyrB 5, infB 5, pps 4.

^e^ 7 alleles matches are found in atpD 3, fusA 3, glnS 3, gltB 5, gyrB 3, infB 201, pps 3.

^f^ 6 alleles matches are found in fusA 1, glnS 3, gltB 3, gyrB 5, infB 5, pps 4.
