# Supplementary material for: Prevalence, Distribution, and Phylogeny of Type Two Toxin-Antitoxin Genes Possessed by Cronobacter Species where C. sakazakii Homologs Follow Sequence Type Lineages
Source: Microorganisms. 2019 Nov 12;7(11):554. doi: 10.3390/microorganisms7110554 (PMC6920972; doi:10.3390/microorganisms7110554)
Supplement: Supplementary file 1 [file microorganisms-07-00554-s001.zip › Supplement Tables 1-3/supplementary figure 1.docx]

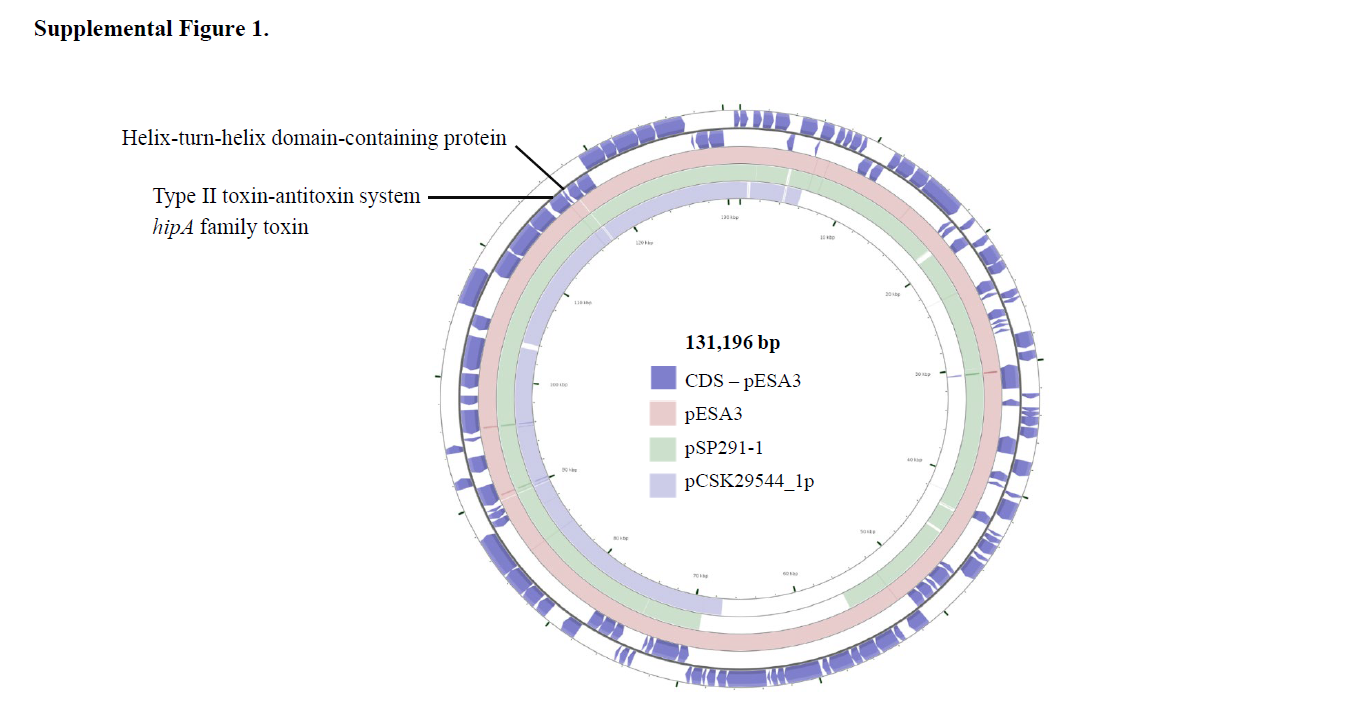


**Supplemental Figure 1.** Alignment of three *C. sakazakii* virulence plasmids, pESA3, pSP291-1, and pCSK29544_1p produced by using CGView Server from the Stothard Research Group (<http://stothard.afns.ualberta.ca/cgview_server/>; last accessed June 25, 2018) showing the alignment of *hipA* and its antitoxin *xre* among the three *C. sakazakii* virulence plasmids The software uses BLAST analysis to illustrate homology among the toxin homologs as well as presence of conserved or the absence of missing homologs. Two circular plasmids– pSP291-1 (NC_020263) and pCSK29544_1p (NZ_CP011048) – were compared against reference plasmid, pESA3 (NC_009780). *hipA* family toxin and a Helix-Turn-Helix (HTH-XRE) domain antitoxin (originally identified as XRE-family transcriptional regulator) are shown.
